# Electrophysiological phenotyping of left ventricular noncompaction cardiomyopathy in pediatric populations: A systematic review

**DOI:** 10.14814/phy2.16029

**Published:** 2024-04-29

**Authors:** Lindsey A. Fitzsimons, Delanie M. Kneeland‐Barber, Gracie C. Hannigan, David A. Karpe, Lyman Wu, Michael Colon, Jess Randall, Kerry L. Tucker

**Affiliations:** ^1^ Department of Biomedical Sciences, College of Osteopathic Medicine University of New England Biddeford Maine USA; ^2^ Albany Medical Center Albany Medical College Albany New York USA; ^3^ Department of Pediatrics Albany Medical College Albany New York USA; ^4^ Pediatric Cardiology, Capital District Pediatric Cardiology Associates Albany Medical College Albany New York USA

**Keywords:** arrhythmia, cardiomyopathy, electrocardiogram, fetal myocardium, honeycomb myocardium, hypertrabeculation, left ventricular noncompaction, noncompaction myocardium, pediatric, spongy myocardium

## Abstract

Left ventricular noncompaction cardiomyopathy (LVNC) is a structural heart defect that has been associated with generation of arrhythmias in the population and is a cause of sudden cardiac death with severe systolic dysfunction and fatal arrhythmias. LVNC has gained increasing acknowledgment with increased prevalence. We conducted a systematic review of reported electrocardiogram (ECG) results for pediatric LVNC patients. EMBASE database query was performed, yielding 4531 articles related to LVNC between 1990 and December 2023. Patient age ranged from prenatal to 18 years of age. Qualitative analyses were performed to characterize individual arrhythmias, and summative interpretation of ECG evaluations was gathered for the entire cohort. Systematic review of 57 LVNC cases and ECG presentation revealed many waveform consistencies, including abnormal left ventricular, atrioventricular node, and interventricular septal patterns, and specifically a high incidence of Mobitz type II and Wolff–Parkinson–White waveforms. This review of ECG analysis reinforces the clinical and etiologic significance of pediatric LVNC. While LVNC in pediatric populations may not always present as acute clinical cases, further investigation into the electrophysiology of the disease supports the need for further evaluation and risk stratification for patients with suspected LVNC and/or ventricular arrhythmia.

## 1 INTRODUCTION

**FIGURE 1:**
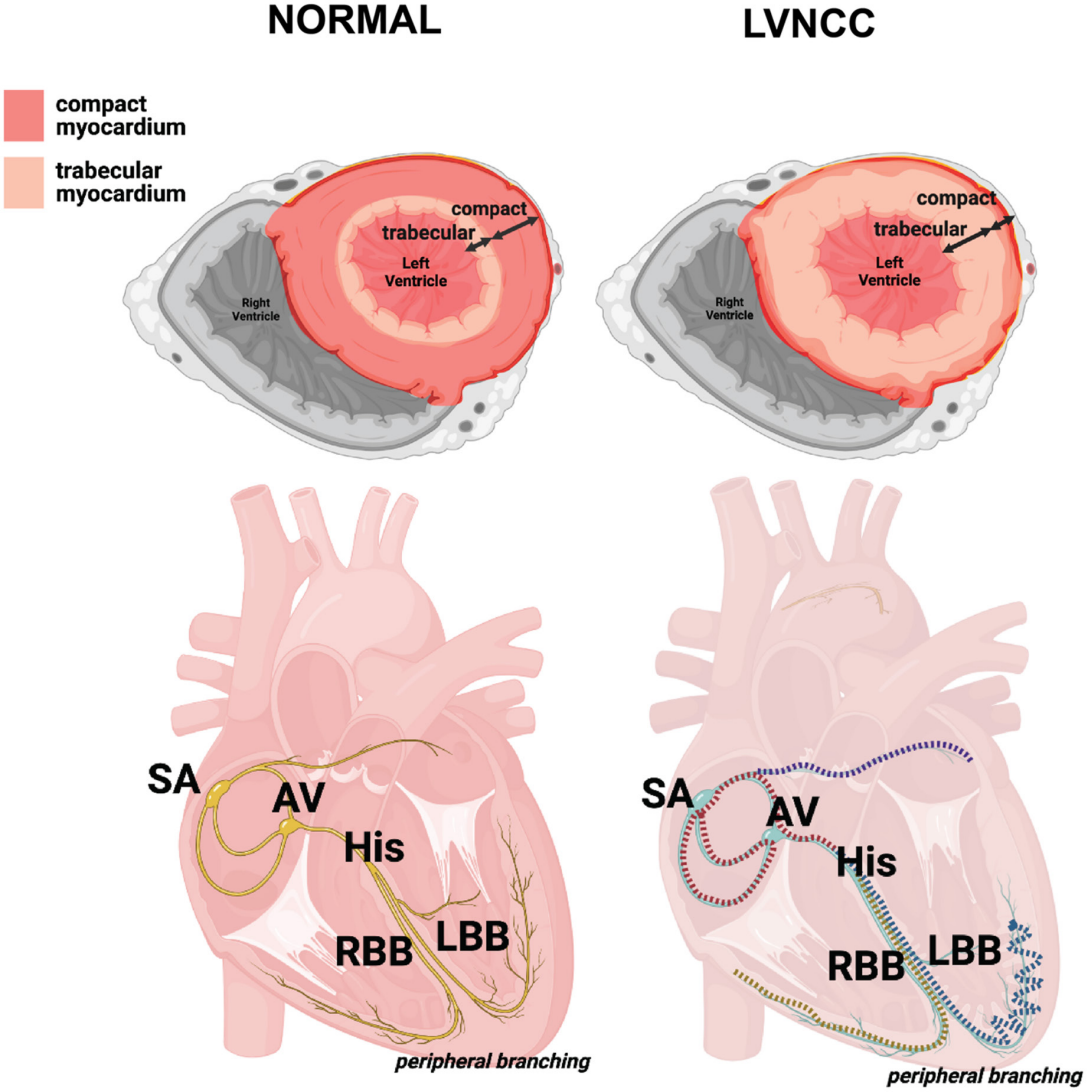
Ventricular morphology and the intrinsic cardiac conduction system in normal humans and LVNC patients. (Left panel, top) Trabecular formation in the healthy, postnatal left ventricle is minimal and represents the spongy interface between the endocardial and myocardial layers of the ventricle. The trabecular myocardium is externally surrounded by the compact myocardial layer, comprising densely packed and tightly organized, force‐producing ventricular muscle. (Right panel, top) LVNC is primarily characterized by excessive trabecular thickness and/or over‐pronounced trabecular recesses that compose the majority of the thickness of the ventricular wall when compared to the thickness of the compact myocardium of the ventricular wall. (Left panel, bottom) The intrinsic cardiac conduction system (iCCS) in a normally developed, non‐diseased human heart is activated at the sinoatrial (SA) node, located in the superior aspect of the right atrium of the heart. Depolarization through the iCCS continues medially from the SA node to the atrioventricular (AV) node and continues into the interventricular septum via the bundle of His, where the electrical activation bifurcates inferiorly and laterally through the RBB and LBB of the ventricular portion of the heart. (Right panel, bottom) Generalized overview of iCCS in LVNC, where the aberrations in peripheral branching can reflect the more jagged, discontinuous distribution of conductive ventricular fibers.

Congenital heart defects (CHD) are the most common of all birth defects, affecting 10.8 per 1000 live births in the United States (Egbe et al., 2014). Left ventricular noncompaction cardiomyopathy (LVNC) is characterized by abnormal trabeculations of the left ventricle, thickening of the left ventricular myocardium consisting of an inner noncompacted and an outer compacted layer, and intertrabecular recesses (Towbin et al., 2015) (Figure 1). LVNC anatomical malformations are diagnostically characterized by excessively thick trabecular extensions and/or exaggerated depth of trabecular recesses that exceeds that of the compact myocardial portion of particularly the apex of the left ventricular chamber wall. Long‐term survival of LVNC patients is reduced (Vaidya et al., 2021), caused by thromboembolism, severe systolic dysfunction, and fatal arrhythmias (Udeoji et al., 2013). However, patients with a preserved left ventricular ejection fraction and normal apical myocardial architecture enjoy lifespans comparable to the general population (Vaidya et al., 2021).

Extensive research has gone into understanding the mechanisms and genotypic spectrum associated with LVNC, which is estimated to affect approximately 2% of pediatric patients with known CHD (Hughes et al., 2007). Conversely, one study (Stähli et al., 2013) estimated that 12% of patients with LVNC diagnosed through standard criteria (Chin et al., 1990; Jenni et al., 2001) display additional CHD, most prominently obstruction of the left ventricular outflow tract and Ebstein's anomaly. LVNC is associated with many genetic mutations, but the causality had been difficult to disentangle because of the diversity of patient phenotypes (Finsterer et al., 2017). While LVNC is congenital, it is not agreed upon whether it is to be considered a distinct cardiomyopathy that stands alone as a CHD (Elliott et al., 2008; Maron et al., 2006). In addition, the exact diagnostic criteria of LVNC are still debated and have not yet been established as standard (Elliott et al., 2008; Floria et al., 2014; Jefferies, 2021; Maron et al., 2006; Oechslin & Jenni, 2011; Ross & Semsarian, 2018; Sharain & Anavekar, 2020). Thus, LVNC represents a diagnosis with a broad spectrum of disease categorizations, and this potentially leads to an undercount of the incidence of CHD in the general population.

The phenotypic presentation of structural abnormalities in the heart can affect electrophysical pathways within the myocardium, manifesting with atrial and ventricular arrhythmias (Karunamuni et al., 2014). LVNC's association with ventricular arrhythmias suggests a role for altered cardiac action potential conduction (Samsa et al., 2013), with respect to the complexity of proper development of the cardiac conduction system and the involved coordination with the myocardial differentiation process (Rentschler et al., 2001; Samsa et al., 2013). Correlating with the proximity of the conduction system's anatomical location within the ventricular wall in early embryonic development, the Purkinje fiber network is impacted by altered ventricular wall maturation (Samsa et al., 2013) (Figure 1), combined with highly specific temporal and genetic regulation of specification and patterning (Burnicka‐Turek et al., 2020; Hatcher & Basson, 2009). This anatomical change within the myocardium then contributes ventricular depolarization via the conduction system, and patients with LVNC are at increased risk of bradycardia, Wolff–Parkinson–White, and other arrhythmias in adults (Miyake & Kim, 2015). Systematic reviews of pediatric cases of LVNC are few in number (Luczak‐Wozniak & Werner, 2021; Thareja et al., 2022), and few articles have summarized ECG findings in either adult or pediatric patients (Bazoukis et al., 2022; Bhaskaran et al., 2021; Czosek et al., 2015; Luczak‐Wozniak & Werner, 2021; Miyake & Kim, 2015; Stollberger & Finsterer, 2010). This project aims to evaluate pediatric cases and ECG findings of this disease while discussing stipulations surrounding diagnosis.

## 2 METHODS

### 2.1 Study design and selection of articles

**FIGURE 2:**
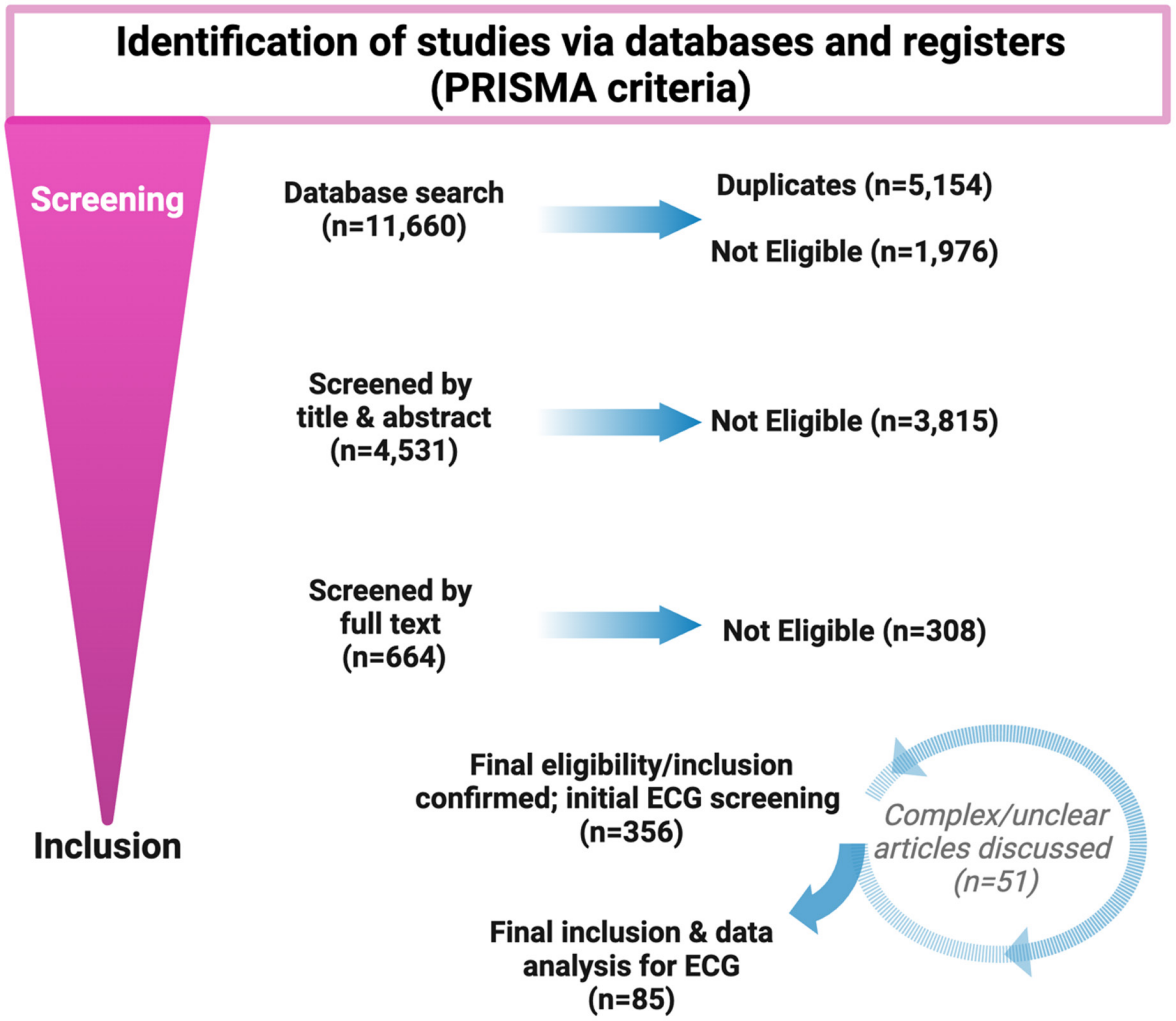
Schematic overview of article and case inclusion and exclusion criteria. See Methods text for details.

PRISMA 2020 guidelines (Page et al., 2021) were used to guide an Embase database search for articles covering LVNC and ranging between October 1990 and December 2023 (Figure 2). The following combination of key terms related to LVNC were implemented as follows: honeycomb myocardium, hypertrabeculated, hypertrabeculation, left ventricular noncompacted, left ventricular noncompaction, left ventricular noncompacted, left ventricular noncompaction, noncompacted cardiomyopathy, noncompacted myocardium, noncompaction cardiomyopathy, noncompaction myocardium, noncompacted cardiomyopathy, noncompacted myocardium, noncompaction cardiomyopathy, noncompaction myocardium, and spongy myocardium. Published studies from 1990 to the search date December 2023 were included. Right sided ventricular hypertrabeculation was excluded in this project because of its limitations with diagnosis and is referred to in greater detail within the limitations of this study (Sato et al., 2007).

The first selection of publications was sifted using EndNote, which deleted all duplicates. Each publication's title and abstract were then reviewed by two independent investigators for determination on qualification for inclusion based on criteria listed above. Excluded studies included editorials, guidelines, expert reviews, letters to journal/editors, books, reviews, clinical findings complicated by dominating cardiomyopathies other than LVNC, non‐human studies, cases in pregnant women or other complex physiological states, and cases involving adult patients. Studies were also excluded if there was no relation to the topic discussed here, inappropriate study design or analysis, if apparent biases were noted, and if LVNC diagnostic criteria were not clearly stated.

**FIGURE 3:**
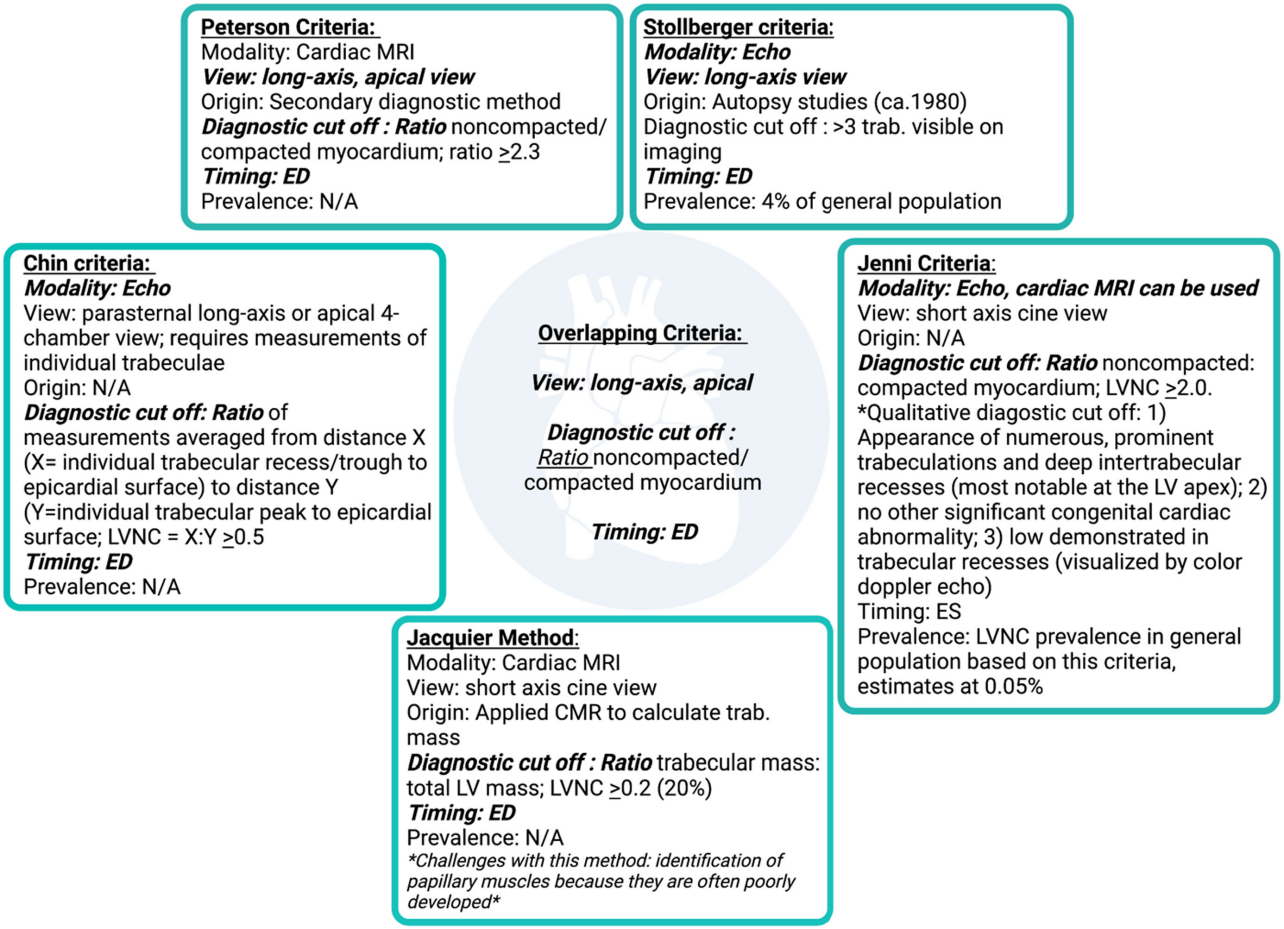
Overview of known clinical and anatomical diagnostic criteria for LVNC. See Methods and Discussion text for details.

In order to meet inclusion criteria, articles to be considered for the review were required to meet or exceed the following criteria:

1. Articles included human subjects
2. Articles were written in the English language
3. Articles patient populations addressed in articles were required to fall within the pediatric/gestational age range up to 18 years of age and 11 months
4. Manuscripts were required to have diagnosed left ventricular noncompaction cardiomyopathy published in a setting where the diagnostic criteria were clearly stated and adhered to. Echocardiograms are the more widely available and are therefore the most commonly used in diagnosis. The three most widely used criteria that utilize echocardiograms are the Chin, Jenni, and Stollberger criteria (Chin et al., 1990; Jenni et al., 2001; Stöllberger et al., 2013) (Figure 3). The Chin criteria establish an X:Y ratio of the hypertrabeculated tissue, where X is the distance from the bottom of the trough to the epicardial surface and Y is the distance from the top of the peak to epicardial surface (Chin et al., 1990). A ratio greater than 0.5 indicates LVNC. The Jenni criteria are currently the most validated and employ echocardiogram to measure the thickness of the noncompacted and compacted myocardium (Jenni et al., 2001). A noncompacted:compacted ratio greater than or equal to 2 is the first requirement for diagnosis. The three additional requirements include the presence of numerous, excessively prominent trabeculations and deep intertrabecular recesses (most notably at the LV apex), no other significant congenital cardiac abnormality, and flow demonstrated in trabecular recesses, as visualized by color Doppler (Jenni et al., 2001). The final echocardiogram criteria come from Stollberger et al. which indicates a diagnosis of LVNC when greater than three prominent trabeculations are present on imaging (Stöllberger et al., 2013). However, only 33% of the studies adhered to one of these published diagnostic criteria (Chin et al., 1990; Jacquier et al., 2010; Jenni et al., 2001; Petersen et al., 2005; Stöllberger et al., 2013).
5. Finally, articles to be included were required to present electrophysiological data from LVNC cases in qualitative and quantitative forms, where a physical 12‐lead ECG present in those cases was included for summative ECG analysis. The final selection of inclusion articles was done by full‐text reading of articles (*n* = 664). Finally, articles were evaluated on eligible 12‐lead ECG presented. A total of 356 studies met inclusion criteria and were included in the analysis.

### 2.2 ECG interpretation and analysis

A total of 85 representative ECG strips and/or specific waveform images were extracted from 52 eligible publications and compiled. These ECG studies were then blinded and partially de‐identified to include patient's age, gender, and symptoms necessitating ECG before being presented to multiple practicing physicians, senior medical students, and an exercise physiologist, all with clinical experience and/or a scientific background in pediatric congenital heart diseases and electrocardiography. Intra‐observer qualitative and semi‐quantitative results were compiled and are summarized in Table 1. Individual cases were categorized by pediatric age group and sex. Diagnostic criteria and ECG outcomes were categorized by etiologic and anatomical origin, as described in the legend for Table 1.

**TABLE 1:** Summary of descriptive and ECG outcomes in pediatric LVNC cases.

		LVH	RVH	Septal	Vtach/Vfib	WPW	Sinus	Multiple	Other
Total number		11	5	12	12	5	6	4	2
Age	Newborn‐12 Months	4	2	3	3	2	1	2	1
	13 Months–12 Years	4	2	2	3	1	3	0	1
	13 Years–18 Years	3	1	7	6	2	2	2	0
Sex	Female	3	4	6	6	4	5	1	0
	Male	7	1	6	6	1	1	3	2
Ventricle	Left	11	4	11	11	4	5	3	1
	Biventricular	0	1	1	1	1	1	1	1
Anatomical Location	Apex	2	1	5	6	4	1	3	0
	Anterior wall	1	0	0	0	0	0	0	0
	Lateral wall	1	0	3	3	1	0	3	0
	Septal wall	1	0	2	0	1	0	1	0
	Posterior wall	1	0	2	1	0	0	1	0
	Inferior wall				2	1	0	0	0
	Base	0	0	0	1	0	0	0	0
**Diagnostic criteria**	Jenni	0	1	2	2	2	1	1	0
	Chin	1	0	0	1	0	1	0	0
	Stollberger	0	0	0	0	0	0	0	0
	Petersen	1	0	2	5	0	0	0	0
	Jacquier	0	0	0	0	0	0	0	0
	Other	9	4	8	4	3	3	3	2
Lvef (%)	≤20%	0	1	1	1	0	0	1	0
	21–30%	0	0	2	2	0	4	1	0
	31–40%	0	0	0	0	0	1	1	1
	41–55%	1	1	2	0	1	0	0	0
	≥56% (preserved/normal)	3	1	5	4	2	1	0	1
Athlete	During Exercise	1	0	1 (syncope related to exercise)	2	0	1 (symptomatic during sports)	0	0
	Other	10	5	11	9	1	4	4	2
	Ethnicity	None stated	Omani (1)	Caucasian (2)	None stated	African (1), Japanese (1)	None stated	None stated	None stated

*Note*: See Methods and Results text for details.

Abbreviations and explanation of categories used to distinguish ECG data: LVH, left ventricular hypertrophy, ECG pattern consistent with LVH; RVH, right ventricular hypertrophy, ECG pattern consistent with RVH; Septal, septal patterns included etiologies of the atrioventricular (AV) node, bundle of His, and abnormalities of the right and left bundle branches; Vtach/Vfib, waveform patterns consistent with ventricular tachycardia (Vtach), ventricular fibrillation (Vfib), multiple premature ventricular contractions, long‐QT, and incidence of Torsades de Pointes; WPW, Wolff–Parkinson–White pattern, characterized by ECG abnormalities similar to AV heart block, but instead indicating presence of accessory pathways; Sinus, SA node patterns included abnormal P‐wave incidence/frequency, amplitude, duration, and/or morphology, and P‐R interval; the “Multiple” etiology group included cases with multiple, complex ECG abnormalities; and, finally, “Other”, where qualitative interpretations varied, as described in the Methods section under “ECG interpretation and analysis.” LV EF (%), left ventricular ejection fraction, expressed as a percentage.

ECG evaluators participated in a group discussion of each case following submission of individual evaluations of all ECG case studies. Following compilation of a summary of outcomes table for each interpreter (Table 1), evaluators were unblinded to case diagnoses and the outcomes table was examined for individual case and categorical discrepancies between evaluators. In order to assess and quantify any discrepancies in analysis/interpretation of LVNC ECG cases, we performed a statistical analysis of interrater reliability between ECG interpreters (Microsoft Excel for iOS, Version 16.79.1, 2023). Percent agreement between the two ECG interpreters for individual ECG assessment outcomes were retroactively coded with either a “1,” indicating a positive interpretation, or a “0,” indicating a negative interpretation for each of the outcome categories (e.g., LVH ECG pattern). This process was performed for the total number of cases identified in Table 1. Percent interrater agreement was calculated by subtracting Interpreter B's values from those of Interpreter A, calculating the total number of zeroes (i.e., agreed upon interpretations), and subsequently dividing the number of agreed upon cases by the total number of examined cases (e.g., of LVH). Analysis of percent agreement between the two ECG interpreters was performed for composite LVNC ECG case analyses. Composite/overall interrater agreement was calculated as 86.7%, which is considered to exceed the recommended 80% minimum acceptable interrater agreement (Gisev et al., 2013; McHugh, 2012). We also employed a chi‐square statistical analysis (Graphpad Prism 10.0.3 (217), September 2023) comparing individual‐interpreter ECG analyses. Chi‐square analysis revealed no statistically significant difference in the incidence of case disagreements between ECG interpreters (*p* < 0.99). Data for these analyses are available upon request.

Irreconcilable inter‐observer discrepancies were identified in a total of 2 out of the 85 total ECG reads (2%), and both cases involved different interpretations/characterizations of second‐degree heart block. These two discrepancies were not deemed to significantly impact the overall assessments and take‐aways and were both included as a separate category in the compilation table to summarize the collective opinions of all evaluators (Table 1).

## 3 RESULTS

### 3.1 Quantification of ECG phenotyping in pediatric LVNC

As recorded in 57 pediatric cases of LVNC with accompanying ECG data, a total of 85 ECG data sets met inclusion criteria and were included for ECG analysis (27 = Male; 29 = Female; 1 = unspecified). Overall, confirmed human cases of LVNC manifested ECG characteristics consistent with ventricular strain, ventricular hypertrophy, and involvement/impairment of the interventricular septum (Table 1). Six cases (~11%) with ECG abnormalities consistent with disruption to and/or involving the sinoatrial (SA) node were observed in mostly female LVNC patients (*n* = 5). There were five cases (~9%) with ECG morphologies consistent with right ventricular hypertrophy, which was noticeably more common in females (80%; *n* = 4) when compared to males.

Defects in the cardiac conduction system (CCS) included second‐degree atrioventricular block (Mobitz II), delayed ventricular reentry patterning, frequent incidence of premature ventricular contractions, right and left bundle branch blocks, and ventricular escape arrhythmia (Table 1). ECG abnormalities involving the atrioventricular (AV) node and structures within the interventricular septum (IVS; bundle of His, R/L bundle branches) were observed in 21% (*n* = 12) of cases. Incidence between females (50%) and males (50%) was identical. The most common AV defect was second‐degree atrioventricular block (Mobitz type II), which was seen in 11% (*n* = 6) of cases and was more common in male (*n* = 4) than in female (*n* = 2) patients.

The primary ECG abnormalities observed were derived from and/or directly linked to structural and/or electrophysiological aberrancies involving the left ventricle. LV‐derived defects were seen in 40% (*n* = 23) of patients with confirmed LVNC, while 19% of cases (*n* = 11) showed ECG waveform abnormalities consistent with left ventricular hypertrophy (LVH), of which 27% were female (*n* = 3) and 64% male (*n* = 7) patients. Interestingly, of LVNC patients with ECG evidence of LVH, 36% (*n* = 4) of cases were noted in the newborn to 12‐month‐old age category, followed by another 36% of cases observed in the 13‐month‐old to 12‐year‐old age group. Ventricular arrhythmias were noted in 21% (*n* = 12) of ECG cases and were further observed in equal incidence between male (*n* = 6) and female (*n* = 6) patients. In contrast to structural findings like LVH, 50% (*n* = 6) of cases with ventricular arrhythmias were observed in the 13‐ to 18‐year‐old age bracket. The most prevalent of LVNC characteristics was accessory pathway patterning. The incidence of the Wolff–Parkinson–White ECG pattern was seen in 9% of all cases (*n* = 5), with a female predominance of 80% (*n* = 4).

## 4 DISCUSSION

### 4.1 Characterization of clinical findings and outcomes

Multiple interpretations and opinions abound regarding etiology, the status of LVNC as an individual disease entity, and the overall clinical significance of LVNC (Elliott et al., 2008; Floria et al., 2014; Jefferies, 2021; Maron et al., 2006; Oechslin & Jenni, 2011; Ross & Semsarian, 2018; Sharain & Anavekar, 2020). The purpose of this systematic review was not to commit to any single, existing diagnostic approach to LVNC. By contrast, the investigators sought to characterize a representative cohort of human cases of LVNC using a stringent, yet consistent set of criteria designed to rule out as much variability as possible. Our approach to qualitative analysis of LVNC clinical cases was to compare and contrast the electrophysiology (as measured with ECG) resulting from confirmed cases meeting our inclusion criteria. The results from a qualitative review of individual ECG cases confirmed that the electrophysiology resulting from LVNC is represented by a broad phenotypic spectrum, ranging in severity from asymptomatic, sporadic disruptions in cardiac rhythm, to clinically symptomatic, life‐threatening shifts and progression of arrhythmogenicity, as has been reported in other systematic reviews of both adult (Bazoukis et al., 2022; Bhaskaran et al., 2021) and pediatric (Luczak‐Wozniak & Werner, 2021) patients. In contrast to one study in the adult population (Bhaskaran et al., 2021), we observed a robust incidence (46%) of arrhythmias traceable to the cardiac apex, a common area of noncompaction. Similar to a systematic review in adults (Bazoukis et al., 2022) and in pediatric patients (Luczak‐Wozniak & Werner, 2021), we observed an appreciable fraction of malignant arrythmias, including ventricular tachycardia and ventricular fibrillation.

### 4.2 High prevalence of Wolff–Parkinson–White ECG pattern in pediatric LVNC

Nine percent of the patient population analyzed in this review demonstrated an ECG pattern characteristic of Wolff–Parkinson–White syndrome, which is consistent with what has been documented in the general population, including documentation in pediatric cases of LVNC (Howard et al., 2019; Stollberger & Finsterer, 2010). In the hearts of patients with Wolff–Parkinson–White syndrome, there exists an abnormal connection between the normally electrically separated atria and ventricles, with conjugation of the two electrical systems at the AV node and His–Purkinje system (Benson & Cohen, 2017). This accessory pathway directly connects the electrical systems of the ventricles and atria, thus allowing electrical activity to bypass the atrioventricular node, and leading to the premature excitation of the His–Purkinje system. This alternative electrical pathway is congenital in origin and is a response to the failure of the myocardial syncytium at the annulus fibrosis of the AV valves during fetal development (Spicer & Anderson, 2013), which then results in quickened electrical conduction that manifests as shortened PR intervals on ECGs (Chambers et al., 2021). Electrophysiologically, these accessory AV pathways have been mapped in many locations along the AV groove or in the septum (Sealy et al., 1978). These congenital contributions to the pre‐excitatory reentry loops call attention to the embryonic origin and the potential contribution that congenitally developed cardiomyopathies can play into the development of childhood arrhythmias and possibly LVNC.

### 4.3 Variability in the diagnostic criteria of LVNC

Diagnosis of LVNC relies primarily on echocardiographic and cardiac magnetic resonance imaging (cMRI) studies that provide measurable evidence of noncompaction/hypertrabeculated myocardium. Manuscripts for this review were required to have diagnosed left ventricular noncompaction cardiomyopathy using diagnostic criteria that were clearly stated and adhered to. Echocardiograms are the more widely available and are therefore the most commonly used in diagnosis, including the Chin, Jenni, and Stollberger criteria (Chin et al., 1990; Jenni et al., 2001; Stöllberger et al., 2013) (Figure 3). As cMRI has become more available in recent years, new criteria specific to this imaging modality have been presented, including the Petersen criteria (Petersen et al., 2005) and the Jacquier method (Jacquier et al., 2010) (Figure 3). Although we found articles that employed any one of these published criteria, the majority (66%) of the analyzed cases employed diagnostic criteria that utilized individual components of the above‐described diagnostics, but did not restrict themselves or refer to any single method. Ideally, a standardization in diagnostic techniques could allow for a more rigorous diagnosis of LVNC, and for a more precise approach to the utility of ECG for the examination and diagnosis of LVNC.

### 4.4 Challenges with LVNC and differential diagnosis

LVNC was first described in 1931 (McMillan et al., 1931) but it took another half century before isolated LVNC started to be more carefully diagnosed and appear in the medical literature (Chin et al., 1990; Engberding & Bender, 1984; Maron et al., 2006). The development of new diagnostic criteria as well as increased technology of diagnostic equipment has enhanced the capabilities as well as broadened the spectrum of what is considered to be diagnostic LVNC (Captur et al., 2013). As prevalence and number of LVNC cases recognized each year increase, the rate of accuracy and severity of these cases also has the potential to increase. This was the pattern seen while collecting these cases for the review, with increased case number as time progressed and clinicians became more familiar with the cardiomyopathy. This pattern of data collection was represented with a non‐normative distribution with a rightward shift represented over the timeline investigated in this article.

### 4.5 Peripheral cardiac conduction system

LVNC‐associated ventricular disorganization is not restricted to the gross morphology of the ventricle. Rather, the LVNC phenotype also includes a critical, microstructural component. It is known that disruption to the integrity of the sarcomeric structure of cardiomyocytes negatively impacts the electrochemical/mechanical and functional capacity of the ventricular myocardium. These deficits are often accompanied by or lead to development of electrophysiological deficits like ventricular arrhythmia (Li et al., 2005; Sheikh et al., 2009). Although the scope of this article does not include a comprehensive orientation to cardiac electrophysiology, we provide a brief overview of the physiology in order to: (1) Aid in defining the peripheral component of the cardiac conduction system; to (2) emphasize the functional significance of the peripheral cardiac conduction system; and finally, to (3) relate the structure and function of the peripheral cardiac conduction system as is most relevant to the pathogenesis and long‐term impact of LVNC in pediatric populations.

The conduction system of the heart begins in the sinoatrial node located at the junction of the crista terminalis and the opening of the superior vena cava (Figure 1). The impulse spreads through and coordinates atrial contraction, where the impulse then converges at the atrioventricular node, located in the Koch triangle, where propagation is delayed for ventricular filling (Samsa et al., 2013). Just below the AV node, bundle of His segment serves as the focal point for the bifurcation of the right and left ventricular bundle branches (RBB and LBB, respectively), which extend inferiorly/distally through the interventricular septum (IVS) and toward the apex of the heart. As the RBB and LBBs travel and surround their respective ventricular chambers, they branch out further into slower, smaller, and more dispersed conductive fascicles known as the Purkinje fibers. Due to the complexity, plasticity, and independent variability of the Purkinje fiber networks, this component of ventricular conduction has been classified as the peripheral cardiac conduction system. (Harris et al., 2012). The peripheral conduction system is responsible for depolarization of the ventricular myocardium to produce a coordinated, forceful ejection of the ventricular chamber, as blood is pumped upward and out of the heart to systemic circulation.

In the developing mammalian heart, the cardiac conduction system does not mature nor is it fully activated until after the elongation and looping phases of the embryonic heart, when atrial and ventricular chambers have begun to septate, and contraction of these compartments begins to occur independently from one another (Bhattacharyya & Munshi, 2020; Mohan et al., 2018), although some functional specialization is observed already by mid‐gestation (Rentschler et al., 2001). Moreover, the maturation of the anatomical portions of the ventricular chambers is orchestrated concurrently with the development, branching, and maturation of the cardiac conduction system nerve fibers (Miquerol et al., 2010). Although perfusion of the embryonic/fetal tissues is primarily provided by the placenta, the developing ventricles continue to pump and help circulate blood throughout prenatal gestation and are subsequently subjected to variance in environmental, hemodynamic, and/or genetic circumstances therein (Haack & Abdelilah‐Seyfried, 2016; Hove et al., 2003). Thus, it needs to be considered that these highly modified conductive cardiomyocytes have a propensity to be disturbed in the same manner during development that the non‐conductive cardiomyocytes do. Taking this into account, it is logical to consider this a congenital physical developmental cause of clinical arrhythmia presentation in childhood where it is also understood that cardiac chamber maturation and abnormalities in this development will cause cardiomyopathies, cardiac cycle dysfunction, and arrhythmias (Samsa et al., 2013).

### 4.6 Ventricular reentry loops and ventricular arrhythmias

The pathophysiology of ventricular arrhythmias in cardiomyopathy is primarily characterized by the formation of one or more accessory pathways between the ventricles and atria, enabling aberrant reentry patterning through the ventricles that is independent from the otherwise regular and rhythmic cardiac cycle. From a structural perspective, accessory pathways generally initiate the loss of cardiomyocyte cell‐to‐cell coupling, providing the proper substrate for reentry and subsequent ventricular arrhythmia observed in cardiomyopathy (Boukens et al., 2009). In pediatric populations, a more focal mechanism may also contribute to formation of accessory pathways and ventricular arrhythmia. Premature ventricular contractions (PVCs) in patients with cardiomyopathies may be of clinical significance as well, especially if seen with levels of complexity, such as in multifocal or couplet patterns. Frequent PVCs of this nature may be predictors of more malignant arrhythmia and sudden cardiac death; however, it is noted that there are limitations with this due to absence of extensive data (Boukens et al., 2009).

Mechanical contributions to ventricular arrhythmias have historically been described with respect to heart failure, but they also apply in cardiomyopathies which experience changing stresses on the myocardial wall (Katz & Rolett, 2016; Tsuda, 2021), as also seen in LVNC. In heart failure and cardiomyopathies, increased electrophysiologic and electromechanical feedback of myocardial tissue leads to a directly proportional increases in the magnitude of ventricular wall stress and subsequent left ventricular dilation (Katz & Rolett, 2016; Tsuda, 2021). This change in wall dynamic can also be seen in LVNC, where the change in trabeculation can contribute to wall thickness and thus the considerable force of the myocardium (Howard et al., 2019; Tsuda, 2021).

## 5 LIMITATIONS

This systematic review was based on published results and collected data from the respective included studies. There is a notable chance of selection bias for several reasons: (1) Many of these studies in the pediatric population were prompted by athlete and sport physical ECG screenings, introducing a sampling bias to this population pool. (2) Additional selection bias exists because of the fact that articles or case studies for publication often include the best visual ECGs and not the most common clinically seen arrythmias. (3) Another limitation of the study design and article selection is that LVNC does not have a singular and/or consistent combination of agreed‐upon nomenclature in the medical community. While diagnostic criteria are abundant, the authors noted many instances where published, clinical cases met more than one LVNC diagnostic criteria, but were described as cases of “hypertrabeculation,” or “myocardial disarray,” among others. This limitation possesses a challenge in having a common screening methodology for articles published over the timeline stated. The inclusion/exclusion criteria of this systematic review were designed to exclude studies that did not contribute to the study's ECG‐focused design as well as studies with insufficient or incomplete data. This could potentially be overcome in the future with more standardized methods of screening, diagnosis, and documentation of LVNC. (4) A final limitation is represented by the patient population and the selection bias that this creates, with screening and documentation of more severe pediatric cases being the ones most likely documented and published. Desirable of course would be a more ideal data collection from these patients, for example a graded stress test that would more accurately assess heart function. We would note that despite the limitations of the collected data, both blinded ECG readers came independently to similar assessments in over 95% of the cases.

## 6 CONCLUSION

Noncompaction of the ventricular myocardium, and LVNC specifically, is characterized by an anatomical malformation of the left ventricular chamber wall, where the thickness and depth of the trabecular myocardium exceed that of the compact myocardium of the heart. The purpose of this systematic review was to critically dissect clinical pediatric cases of LVNC and further summarize the electrophysiological consistencies observed across this patient population. While the clinical and functional impacts of LVNC as well as the etiology, diagnostic classification, and the primary or acquired disease nature of this condition are widely debated, the coincidence and severity of cardiac arrhythmia, especially malignant ventricular arrhythmia, observed in LVNC patients cannot be ignored. Abnormal ECGs are considered one of the variables to consider along with left ventricular ejection fraction, age, sex, family background, and cardiovascular risk factors to assess clinical risk prediction (Casas et al., 2021).

Clinically speaking, LVNC is often considered to be a compensatory and/or subclinical comorbidity rather than an independent disease with causal factors. Our investigation not only supports LVNC as an independent diagnostic entity but also documents its prevalence in pediatric populations and its clinical relevance beyond simply anatomical changes to the anatomy of the left ventricle. Because LVNC by itself is not typically acutely life‐threatening, patients with “subclinical” LVNC, especially children, display consistent and clinically significant electrocardiographic findings to justify appropriate diagnostic evaluation, risk stratification, and even prophylactic intervention as these patients mature into young adulthood as well as when they continue to age through adulthood. Additionally, we offer a clinical characterizing perspective of ECG in LVNC which can support the inclusion of LVNC to the differential diagnoses and further risk stratification, prior to the need for exercise stress testing and/or expensive cardiac imaging requiring prior authorization.

We provide this systematic review as an effort to substantiate the idea that LVNC is a worthy anatomical phenomenon to study and its associations with arrhythmia deserving consideration for screening and early intervention in pediatric patients. We would also like to call attention to the absence of specific coding for LVNC and how if standard coding were to be implemented, it would not only help to monitor how this condition initially presents, progresses, and comes to fruition, but could also support a more standardized approach to risk stratification for patients with known or suspected LVNC. Because of unclear data and discrepancies with documentation, LVNC progression presents sporadic and is not fully understood. LVNC often begins subclinically and, at some point during the manifestation of disease, transitions into a clinical comorbidity level severe enough to constitute life‐threatening cardiac arrhythmias and risk of sudden death. If proper coding were implemented, this cardiomyopathy would be better understood and perhaps ultimately prevented.

## AUTHOR CONTRIBUTIONS

The study was conceived and designed by Lindsey A. Fitzsimons, Delanie M. Kneeland‐Barber, and Kerry L. Tucker. Lindsey A. Fitzsimons, Delanie M. Kneeland‐Barber, Gracie C. Hannigan, David A. Karpe, and Kerry L. Tucker scanned the literature and reviewed abstracts and full texts as needed. ECG data were harvested and analyzed blind by Gracie C. Hannigan, Lindsey A. Fitzsimons, and Lyman Wu. The data, conclusions, and final manuscript were reviewed by Michael Colon and Jess Randall. The manuscript was written by Lindsey A. Fitzsimons, Delanie M. Kneeland‐Barber, and Kerry L. Tucker. Lindsey A. Fitzsimons prepared all figures. All authors reviewed and approved of the final manuscript before submission.

## FUNDING INFORMATION

The authors gratefully acknowledge the Saving tiny Hearts Society, whose generous financial support allowed this study to be performed.

## CONFLICT OF INTEREST STATEMENT

The authors attest to no conflicts of interest.

## ETHICS STATEMENT

This systematic review was conducted in accordance with the ethical guidelines of the respective institutions of the authors. Any requirement for written informed consent was waived because the data analyzed in this study originated from the primary literature and were thereby anonymized.

## Data Availability

Publicly available DOI for Figshare data: https://doi.org/10.6084/m9.figshare.24763800.
